# Quantifying and communicating the burden of COVID-19

**DOI:** 10.1186/s12874-021-01349-z

**Published:** 2021-08-10

**Authors:** Maja von Cube, Jéan-Francois Timsit, Andreas Kammerlander, Martin Schumacher

**Affiliations:** 1 Institute of Medical Biometry and Statistics, Faculty of Medicine and Medical Center - University of Freiburg, Stefan-Meier-Str. 26, 79104 Freiburg, Germany; 2 UMR 1137 IAME Inserm/Université Paris Diderot, 16 Rue Henri Huchard, 75018 Paris, France; 3 APHP Medical and Infectious Diseases ICU, Bichat Hospital, 46 Rue Henri Huchard, 75877 Paris, France; 4 Institute for Economics, Department of International Economic Policy, University of Freiburg, Rempartstraße 10 - 16, 79098 Freiburg, Germany; 5 Institute of Medical Biometry and Statistics, Faculty of Medicine and Medical Center - University of Freiburg, Stefan-Meier-Str. 26, 79104 Freiburg, Germany

**Keywords:** Preventable deaths, SARS-CoV-2, Population attributable fraction, P-score, Standardized mortality ratio, Excess deaths, Per capita rate, Z-score

## Abstract

**Background:**

An essential aspect of preventing further COVID-19 outbreaks and to learn for future pandemics is the evaluation of different political strategies, which aim at reducing transmission of and mortality due to COVID-19. One important aspect in this context is the comparison of attributable mortality.

**Methods:**

We give a comprehensive overview of six epidemiological measures that are used to quantify COVID-19 attributable mortality (p-score, standardized mortality ratio, absolute number of excess deaths, per capita rate, z-score and the population attributable fraction).

**Results:**

By defining the six measures based on observed and expected deaths, we explain their relationship. Moreover, three publicly available data examples serve to illustrate the interpretational strengths and weaknesses of the various measures.

Finally, we give recommendation which measures are suitable for an evaluation of public health strategies against COVID-19. The R code to reproduce the results is available as online supplementary material.

**Conclusion:**

The number of excess deaths should be always reported together with the population attributable fraction, the p-score or the standardized mortality ratio instead of a per capita rate. For a complete picture of COVID-19 attributable mortality, quantifying and communicating its relative burden also to a lay audience is of major importance.

**Supplementary Information:**

The online version contains supplementary material available at 10.1186/s12874-021-01349-z.

## Background

Mortality attributable to COVID-19 is a fundamental metric to justify and evaluate decision making on preventive interventions [1, 2]. For example, an important aspect in identifying effective emergency response plans employed by different countries is a comparison of the resulting COVID-19 attributable mortality. Attributable mortality refers to the deaths that were preventable had COVID-19 been eliminated from the population [3].

However, differentiating COVID-19 attributable deaths from those expected to have also happened without COVID-19 is a major challenge. Efforts of case-specific judgement whether a death was preventable cannot be performed efficiently on a large basis. Moreover, in several instances where case-specific judgements were attempted it was reported that these were subjective, inconsistent and maybe even politically motivated [4].

Therefore, a population-based statistical approach is a powerful tool to quantify attributable mortality while maintaining objectivity and transparency. The aforementioned severe issues in determining the cause of death by case-specific judgement are avoided and economic efficiency is ensured. A statistical approach for determining COVID-19 attributable mortality includes not only the correct estimation procedure but also the correct choice of epidemiological measure and a communication of the results to a broad audience including clinicians, medical researchers, public health authorities and also journalists [5].

In epidemiological literature, attributable mortality is often used to refer to a specific epidemiological measure that allows for the interpretation as proportion or number of attributable deaths. However, terminology is inconsistent and there are different metrics which are referred to as attributable mortality [6]. In this article, we discuss the epidemiological measures that are used to quantify COVID-19 attributable mortality. Our comprehensive overview includes a discussion of their interpretation and their relationship to each other. Using publicly available data from the United States, Germany, and the Lombardy in Italy, we explain the interpretational strengths and weaknesses of the various measures.

We obtained all of these estimates from all-cause mortality data [7, 8]. This avoids miscounting of COVID-19 deaths from underreporting, is independent of COVID-19 testing capacities and differentiates indirectly between COVID-19 attributable deaths and expected deaths among COVID-19 infected individuals thereby avoiding subjectivity [2]. While direct COVID-19 attributable deaths cannot be identified, excess deaths estimated from all-cause mortality data quantify the total burden of the pandemic [9]. Besides the evaluation of the potential benefit of prevention strategies, this is one main reason for quantifying attributable mortality. Additionally, attributable mortality is often compared between different countries or populations to understand who is most affected by the pandemic. Such comparisons have important political implications which is why we put specific focus on this aspect. Finally, to achieve acceptance of the population wide preventive interventions, estimates of COVID-19 attributable morality must be communicated in an understandable way to a lay audience. Especially in this pandemic, attributable mortality is not only reported by scientific journals, but also by public media. Therefore, we also discuss which measures can be easiest communicated without leading to misconceptions.

## Methods

We performed a literature search on web of science to give an idea of the most commonly reported metrics of COVID-19 attributable mortality. The search terms were “COVID-19” OR “SARS-CoV-2” AND [“attributable deaths” OR “excess deaths” OR “attributable mortality” OR “excess mortality”], search date was October 13, 2020. In total, we found 147 studies out of which 113 were excluded as they did not report or appropriately quantify attributable/excess mortality. Also one study was excluded as it was not in English. Details on the search are available in the online supplementary material.

### Estimands of attributable mortality

Attributable mortality is by definition a causal measure [10]. It quantifies the number or proportion of preventable deaths had the risk of a specific harmful exposure, such as COVID-19, been eliminated. Thus, it compares the mortality risk of a population under two different settings: the factual setting and a counterfactual setting in which the harmful exposure is entirely removed from the population. Attributable mortality should only be estimated if the exposure (e.g. COVID-19) has a causal effect on mortality. If this prerequisite is not met, then the resulting quantity has no meaningful interpretation.

We denote by $O$ the number of observable deaths corresponding to the factual setting. By $E$, we denote the number of deaths that would be expected in the counterfactual setting. In the following, we define the various estimands of attributable mortality using $O$ and $E$ [10]. It should be noted that $O$ and $E$ are themselves estimands. In the next section, we give a brief explanation on how $O$ and $E$, and thereby all the estimands, can be estimated with all-cause mortality data.

The most often reported metric that quantifies attributable mortality is the total number of excess deaths, defined by the difference of observable and expected deaths (risk difference (RD)) [11], i.e.

$$\mathrm{RD} =O-E.$$

The total number of excess deaths is an important public health indicator that shows how many individuals have died due to the pandemic. From a total of 35 articles 20 (74%) reported the RD.

However, the RD depends highly on the size of the population. Therefore, the RD is often accompanied by a per capita rate (7 articles, 20%). The per capita rate standardizes the number of excess deaths to the total population, often to 100,000 inhabitants, i.e.

$$\mathrm{RD_{p.c.}}=\frac{O-E}{n}\times \mathrm{100,000},$$

where $n$ is the size of the population. Aron and Muellbauer [12] propose to report the relative proportion of COVID-19 attributable deaths as the percentage of deaths above the expected level, sometimes referred to as p-score. This metric is given by

$$\mathrm{p}-\mathrm{score} =\frac{O-E}{E}.$$

Directly related to the p-score is the standardised mortality ratio (SMR) [11], which is defined by

$$\mathrm{SMR}=\frac{O}{E}.$$

A total of 21 (60%) articles reported either the p-score (10, 29%) or the SMR (11, 31%).

Another measure that is sometimes used to compare attributable mortality is the z-score (e.g. [13]). A simplified definition is given by

$$\mathrm{z}-\mathrm{score} =\frac{O-E}{sd\left(O\right)},$$

where $sd\left(O\right)$ is the standard deviation of $O$. The z-score indicates to what extend (measured in units of standard deviations) the excess deaths deviate from what would be considered as a ‘normal’ deviation. It can be viewed as a statistic for testing whether the observed deaths significantly exceed the expected deaths $.$ FluMOMO [13] publish z-scores based on weekly death counts. Moreover, the z-score has been reported by two articles (6%).

Finally, a measure that quantifies the proportion of observable deaths that are attributable to COVID-19 is the population attributable fraction (PAF) [10, 11], which is defined by

$$\mathrm{PAF}=\frac{O-E}{O}.$$

Even though in the epidemiological literature it is a widely known, well-investigated measure the PAF has been reported by only one (3%) study.

### A brief comment on estimation

To estimate the six epidemiological measures only $O$ and $E$ must be estimated. Then they can be plugged into the according definitions to obtain estimates of the measures. We highlight that both $O$ and $E$ are theoretical quantities (estimands). The way they are estimated depends on the data situation. In the following, we explain specifically for COVID-19 as exposure a simple ad-hoc approach which uses all-cause mortality counts for estimation. All-cause mortality counts are routinely collected by federal statistical offices.

The number of observable deaths $O$ can be directly estimated by the number of factually observed deaths in the target population. This is simply the sum of the death counts: $O$ is obtained by sequentially accumulating death counts from the beginning of the pandemic (e.g. March 1, 2020 [14, 15]), up to the current date. In practice, often the estimand $O$ is used interchangeably with its estimator $\widehat{O}$. However, these quantities are not the same and should be differentiated.

The number of expected deaths $E$ had COVID-19 been eliminated can be only estimated with strong assumptions. Assuming that the population risk in previous years was the same as in 2020 with the only difference that COVID-19 has not yet spread, $E$ is estimable by the total number of observed deaths in the same time period but from a previous year, where COVID-19 had not yet occurred. The clearly known start of the COVID-19 pandemic and the possibility to account for differences in population size and age structure justify this assumption. Nonetheless, there is a general trend towards a decrease in mortality which is not explainable by changes in population size and age structure alone, but a number of other factors such as improvements in life sustaining treatments and changes in mortality due to noncommunicable diseases. It must be considered that this may lead to an overestimation of $E.$ Moreover, a year should be chosen in which seasonal mortality trends are comparable to those in 2020. These include especially influenza activity and heat waves.

To account for yearly changes in the total population, $\widehat{O}$ and $\widehat{E}$ should be standardized by the size of the population. Furthermore, it is often necessary to account for differences in the age distribution [16]. This can be achieved by using the weighted average of stratified estimates. The age-stratified estimates quantify the attributable burden within the specific age groups. A wide range of other approaches which account for seasonal effects (influenza, heatwaves) are available [17–19]. Due to the circumstance that $E$ is never observed, the aforementioned untestable strong assumption must be made for its estimation. This applies irrespective of the chosen approach and also to methods which are based on the average death counts of multiple years or which account for seasonal effects. If the assumption on the behaviour of the population risk cannot be justified, $E$ can be considered as the observed number of deaths when COVID-19 has not yet occurred. However, the resulting estimates cannot be interpreted causally as number/proportion of attributable deaths. Instead, the approach only *describes* how death counts have changed in 2020 compared to previous years. We highlight that such a descriptive approach is not sufficient for evidence based decision making on prevention strategies. The descriptive interpretation provides only limited information on the burden of COVID-19. Therefore, the validity of the causal assumptions should be discussed when estimating attributable mortality.

### Analytic approach for the data examples

In this article, we focus on the total burden of the pandemic and therefore consider $O$ and $E$ as cumulative measures over calendar time. It is also common to report weekly numbers of excess deaths. The aim of these studies is primarily detection of outbreaks [7, 20].

To illustrate interpretation and for comparison of the measures, we present three real public data examples. First, we estimate the RD, the RD_p.c._, the SMR, the p-score and the PAF by using public estimates of excess deaths in specific states of the United States [15]. Here expected deaths were quantified using the Serflings model [17] on all-cause mortality data from previous years (January 5, 2015, to January 25, 2020). The Serflings model includes seasonal effects that account for differences in influenza activity [15, 17, 21]. With the estimates of observable and expected deaths, the five measures can be obtained in a straightforward way by plugin of $\widehat{O}$ and $\widehat{E}$ into the formulas.

Second, we use all-cause mortality data from the German Federal Statistical Office [22]. We estimate the p-score, the SMR and the PAF over calendar time using the year 2016 as reference year to estimate the number of expected deaths. In the year 2020 influenza activity was low and comparable to influenza activity in 2016. Stang et al. [23] estimated $E$ as the average of deaths between year 2016 and 2019. However, as influenza activity was high in 2017 and 2018 with many deaths, we believe that 2016 is a better proxy for the number of expected deaths than the average over the previous four years [24, 25]. We take the 10^th^ calendar week (beginning on March 2, 2020) as starting point of the pandemic [23]. To account for changes in age distribution and to understand differences in burden for different age groups, we stratify by age with a cut point at 65 years.

Finally, we use published mortality data of the local community Nembro, in the Lombardy, Italy, to compare the p-score and the PAF and to discuss the z-score. The estimates are stratified by sex [26]. The analysis of the published death counts from Nembro is performed without additional tools.

## Results

First, we discuss the relationship of the epidemiological measures by comparing the corresponding estimands. Our way of defining the estimands shows that all measures depend on $O$ and $E.$ The p-score and the PAF are percentages with RD in the numerator and $E$ or respectively $O$ in the denominator. The RD_p.c._ is also based on RD but has the population size $n$ as denominator. While SMR is a factor composed of $E$ and $O$, it has a straightforward relationship with the p-score by SMR-1 = p-score. Thus, the SMR and the p-score result in basically the same interpretation. The SMR can be interpreted as a percentage that corresponds to the percentage quantified by the p-score. Assuming the death counts to be poisson distributed, the relationship between the z-score and the p-score becomes obvious when considering that $sd\left(O\right)=\sqrt{E}$.

As mentioned in the introduction, COVID-19 attributable mortality is used to quantify the burden of the pandemic and the potential benefit of preventive interventions. Moreover, attributable deaths are compared between different countries and populations to evaluate who is most affected. We now discuss the measures with respect to these aspects.

All of them allow for conclusions on the burden of the pandemic with respect to mortality. When it comes to quantifying the potential benefit of preventive interventions and to a comparison of attributable mortality between different populations some of the measures are more suitable than others. The z-score differs from the other five measures by not directly quantify attributable mortality as total or relative number of deaths. Therefore, it will be discussed separately at the end of this section.

The RD informs on the total number of individuals whose death was attributable to COVID-19. As the total numbers of observable and expected deaths depend on the population, the RD itself cannot be directly generalized to populations of different sizes. For decision making on preventive interventions, such a generalization is often necessary. This is because evidence on their potential benefit is based on the past, while decisions are made for the prevention of deaths in the future. The dependency on the population size also hampers a fair comparison of attributable mortality. Populations of a larger size have naturally a higher number of observable and expected deaths leading to a higher number of excess deaths.To overcome these limitations, often the per capita rate RD_p.c._ is reported [14, 15, 27]. The RD_p.c._ provides information on the total number of attributable deaths per 100,000 in habitants. Thus, the generalization to populations of different sizes is achieved. However, the RD is only indirectly influenced by the population size. The quantities that have a direct influence on the RD are the number of observable and expected deaths. For example, an elderly population has a higher number of expected deaths which leads naturally to a higher number of excess deaths irrespective of the population size. While the RD_p.c._ takes only the population size into account, the SMR, the p-score and the PAF take the number of observable or respectively expected deaths into account and are therefore more suitable to quantify the relative burden of COVID-19.

The following fictional and real data examples illustrate this point.

The fictional example (Table 1) shows how severely misleading a comparison of attributable deaths based on RD and RD_p.c._ may become. We consider two populations (A and B) each having the same number of observed ($\mathrm{O}=1000)$ and expected deaths ($\mathrm{E}=900)$ and thus the same number of excess deaths ($\mathrm{RD}=100)$. The only difference is that population B is larger than population A (500,000 versus 100,000 people). This results in RD_p.c._ = 100 in population A and RD_p.c._ = 20 in population B. However, this difference is not explained by the attributable burden of COVID-19, but simply by the fact that population A has a higher baseline mortality than population B (0.9% versus 0.18%). The example demonstrates that RD_p.c._ is not adequate for a comparison of attributable deaths.

**Table 1:** Fictional data example. ${\varvec{E}}$ expected deaths, ${\varvec{O}}$ observed deaths, ${\varvec{R}}{\varvec{D}}$ total number of excess deaths, ${{\varvec{R}}{\varvec{D}}}_{{\varvec{p}}.{\varvec{c}}.}$ per captia rate, ${\varvec{S}}{\varvec{M}}{\varvec{R}}$ standardized morality ratio, ${\varvec{P}}{\varvec{A}}{\varvec{F}}$ population attributable fraction

Definition	Population A (high baseline mortality)	Population B (low baseline mortality)
$E$	$900$	$900$
$O$	$1000$	$1000$
$n$	$\mathrm{100,000}$	$\mathrm{500,000}$
$RD$	$100$	$100$
${RD}_{p.c.}$	$100 per \mathrm{100,000}$	$20 per \mathrm{100,000}$
$p-score$	$11.1\%$	$11.1\%$
$SMR$	$1.1$	$1.1$
$PAF$	$10\%$	$10\%$

**Fig. 1:**
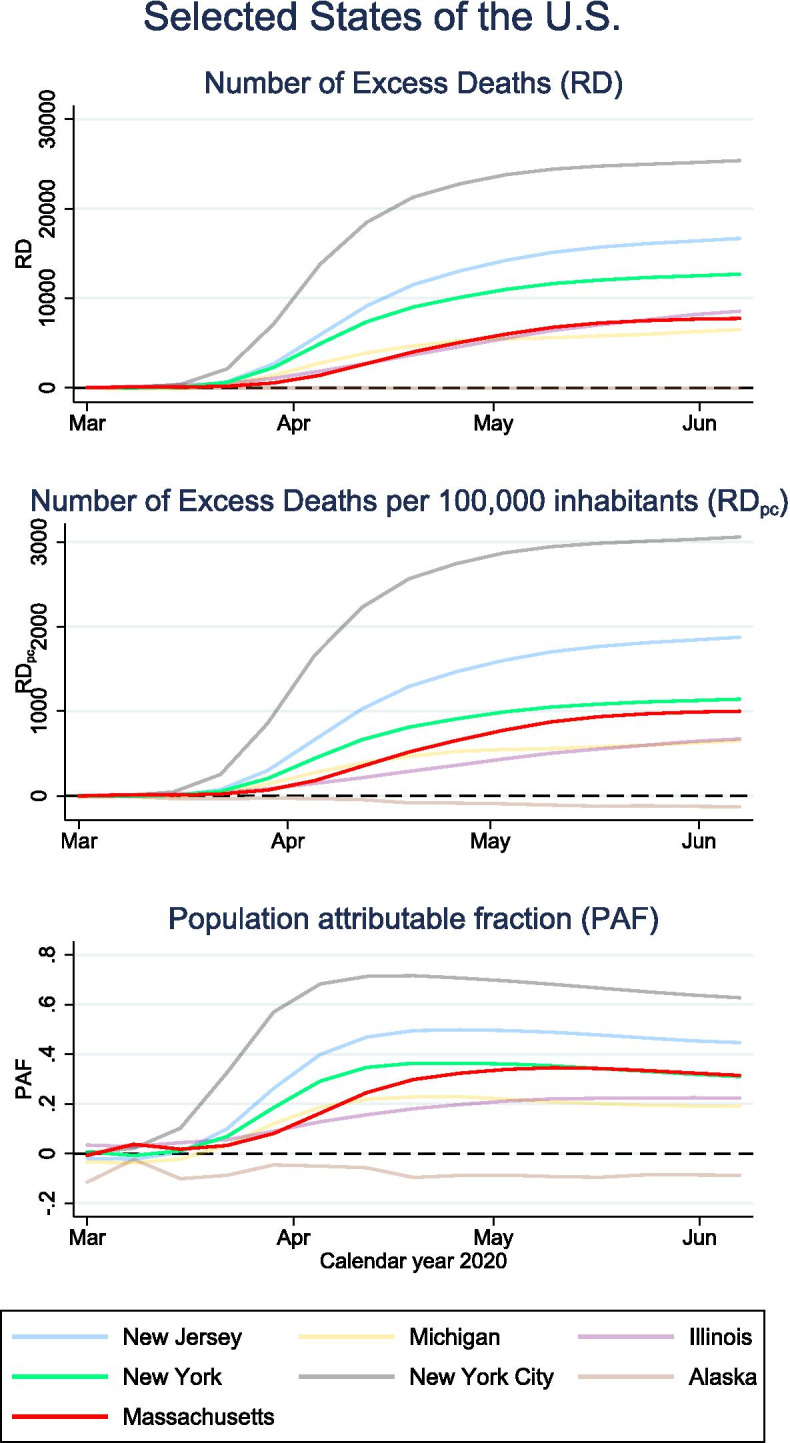
The upper panel shows the cumulative excess risk (RD) over calendar time for states with a large number of reported COVID-19 deaths. The panel in the middle shows the excess deaths per 100,000 inhabitants (RD_p.c._). The lower panel shows the population attributable fraction (PAF). The data was obtained from [15]. The number of expected deaths had COVID-19 been eliminated is an adjusted average over previous years using the Serflings model [17]

Our second example is based on real data from the U.S. [15]. Figure 1 shows the RD over calendar time for selected states of the U.S. (first panel), the RD_p.c._ (second panel) and the PAF (third panel). Since the SMR, the p-score and PAF exhibit the same interpretational benefit compared to RD and RD__p.c_, we only show the results for PAF. We present the results for states with a large number of reported COVID-19 deaths and for Alaska as reference for a state that was barely affected (on May 30, there were 9 COVID-19 related deaths reported [14, 15]).

On June 13, 2020, New York (excluding New York City) had almost twice as many excess deaths than Massachusetts (RD = 12,684 and 7,732 respectively, first panel) and 14% more excess deaths per 100,000 inhabitants (RD_p.c._ = 1142 and 1000 respectively, second panel). Nonetheless, the relative burden of COVID-19 was the same with PAF = 31% attributable deaths in both states (third panel).

The RD, the RD_p.c._ and the percentages SMR, p-score and PAF all quantify attributable mortality, but at different scales. The above examples shows not only that the relative measures allow for important insights that cannot be captured by the RD and RD_p.c._ but also that reporting of RD and RD_p.c._ alone may be severely misleading. For evaluation of the potential benefit of prevention strategies and comparison of attributable deaths between different populations, the RD should be either related to the number of expected deaths (SMR, p-score) or the number of observed deaths (PAF).

**Fig. 2:**
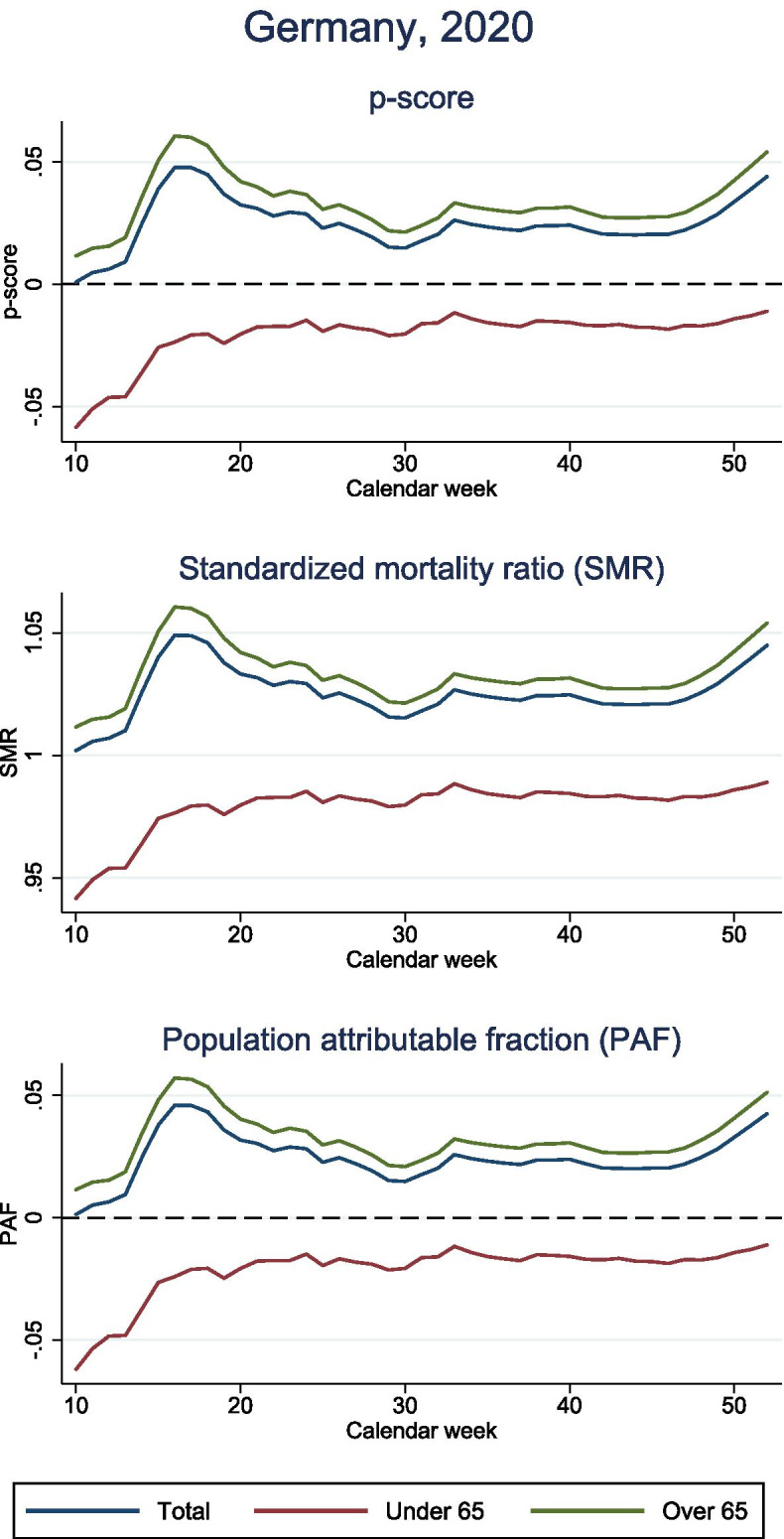
P-score, standardized mortality ratio (SMR) and population attributable fraction (PAF) of the COVID-19 pandemic in Germany adjusted for age and stratified by age group. The expected deaths had COVID-19 been entirely eliminated correspond to the number of observed deaths within the same time period, but from the year 2016

Based on data from the federal statistical office in Germany [22, 23], we illustrate the SMR, the p-score and the PAF (Fig. 2). Thanks to a delay of the outbreak in Germany, preventive interventions could be implemented early and shortage of health care capacities was circumvented. In Fig. 2, we display the three measures for the total German population as well as for people older and younger than 65. There was no attributable mortality among people younger than 65. In contrast, the preventive interventions seem to have decreased all-cause mortality to a level lower than usual. This could imply that the lock down prevented deaths from other causes among younger people. Among individuals aged over 65 5.1% of all observed deaths in 2020 were directly or indirectly attributable to COVID-19. In total, this led to a COVID-19 attributable mortality of 4.2%.

As SMR, p-score and PAF all depend on $O$ and $E$, the behaviour over time is proportional. Thus, all three measures allow for the same conclusions on attributable mortality. The only difference is in their interpretation. While the p-score quantifies the proportion of deaths that are above the expected level, the PAF quantifies the proportion of excess deaths among all observed deaths. The latter is commonly interpreted as proportion of preventable deaths had COVID-19 been eliminated. The SMR is reported as a factor that quantifies to what extend excess deaths are above the expected level. For communication, the SMR is often translated to the p-score.

In the following, we compare the p-score and the PAF. The PAF explains the number of observed deaths. A PAF of 100% means that all observable deaths were preventable, while a PAF of zero means that none of the deaths were preventable. Thus, the PAF does not exceed 100% by definition.

The p-score uses the number of expected deaths as reference. A p-score of zero implies that there are no deaths above the expected level. In contrast to the PAF, the p-score can be become arbitrarily large. Thus, there is no orientation for a maximal value. Differences between the p-score and the PAF are also illustrated by the following example.

Using the published death counts of the Italian study [26], we estimate the p-score and the PAF for women and men from Nembro in the Lombardy, which was one of the most severely affected regions in Europe [26]. The results are shown in Table 2 A. Between January 1, 2020 and April 4, 2020, there were 95 reported deaths among men aged 65 or older and 80 among women aged 65 or older. This was in clear contrast to the previous four years, when mortality ranged between 9 and 16 cases for men and 14 to 25 for women. Both sexes were severely affected by the pandemic. Yet, there were 69% more observed deaths among women than among men. Estimation of the p-score shows that men were with 631% of deaths above the expected level clearly more affected than women with 264% deaths above the expected level. We come to the same conclusion with the PAF. Here we find that 86% of all observed deaths among men were attributable to COVID-19 and 73% of the observed deaths among women. We can conclude that 14% (27%) of the observed deaths among men (women) were not attributable to COVID-19.

**Table 2:** A: Results of the p-score and the population attributable fraction (PAF) on April 4, 2020, for the population older than 65 in Nembro, Italy, stratified by gender. The number of deaths was obtained from [26]. The death counts are not related to the size of the total population and estimates are therefore just approximations. The number of observed deaths until April 4, 2020, is 95 (${{\varvec{O}}}_{{\varvec{m}}}$) among men and 80 (${{\varvec{O}}}_{{\varvec{f}}}$) among women. B: Results of the z-score for the real Nembro population, and a population with the same mortality pattern but 100 times the size of the Nembro population

**A: Results of the p-score and the population attributable fraction (PAF)**								
Reference year	Expected deaths		RD		p-score (in %)		PAF (in %)	
	Men (${E}_{m}$)	Women (${E}_{f}$)	Men (${O}_{m}-{E}_{m}$)	Women (${O}_{f}-{E}_{f}$)	Men $\frac{\left({O}_{m}-{E}_{m}\right) }{{E}_{m}}$	Women $\frac{\left({O}_{f}-{E}_{f}\right) }{{E}_{f}}$	Men $\frac{\left({O}_{m}-{E}_{m}\right) }{{O}_{m}}$	Women $\frac{\left({O}_{f}-{E}_{f}\right) }{{O}_{f}}$
2016	16	14	79	66	493.8	471.4	83.2	82.5
2017	9	24	86	56	955.6	233.3	90.5	70.0
2018	13	24	82	56	630.8	233.3	86.3	70.0
2019	14	25	81	55	578.6	220.0	85.3	68.8
Mean	13	22	82	58	630.8	263.6	86.3	72.5
**B: Results of the z-score**								
Reference year	Expected deaths (population size n)		RD (population size n $\times$ 100)		z-score (population size n)		z-score (population size n $\times$ 100)	
	Men (${E}_{m}$)	Women (${E}_{f}$)	Men (${E}_{m}$)	Women (${E}_{f}$)	Men $\frac{\left({O}_{m}-{E}_{m}\right) }{\sqrt{{E}_{m}}}$	Women $\frac{\left({O}_{f}-{E}_{f}\right) }{\sqrt{{E}_{f}}}$	Men $\frac{\left({O}_{m}-{E}_{m}\right) }{\sqrt{{E}_{m}}}$	Women $\frac{\left({O}_{f}-{E}_{f}\right) }{\sqrt{{E}_{f}}}$
2016	16	14	1600	1400	19.8	17.6	198	176
2017	9	24	900	2400	28.7	11.4	287	114
2018	13	24	1300	2400	22.7	11.4	227	114
2019	14	25	1400	2500	21.6	11.0	216	110
Mean	13	22	1300	2200	22.7	12.4	227	124

The data example shows that the p-score can take large values. For the reference year 2017, it becomes 955%.

In contrast, the PAF is 90.5%. Knowing that 100% is the maximal value, the PAF indicates that almost all deaths observed in 2020 are attributable to COVID-19, if 2017 is used as the reference year.

Finally, we discuss the z-score. Generally, the z-score is used to compare observations that come from different normal distributions. However, the z-score is also used to detect outbreaks and to compare mortality patterns between different populations [13, 28]. In the context of comparing cumulative attributable deaths the z-score has some major limitations. First, it does not quantify the number of proportion of attributable deaths itself. Its interpretation as number of standard deviations away from what is expected cannot be easily communicated to a lay audience.. Second, it allows only for a good comparison of variables that are drawn from a normal distribution. The number of observed (weekly) deaths are not normally distributed, instead they are commonly assumed to be poisson distributed [28, 29]. Under the poisson distribution the z-score becomes

$$\frac{O-E}{\sqrt{E}}$$

Thus, the z-score depends on the population size, as the standard error is a function of the expected value. Due to this dependence, attributable deaths from countries with large differences in population size are flawed as can be easily seen from the data example shown in Table 2 B. We consider again the published death counts of the Italian study [26]. We estimate crude z-scores (not accounting for overdispersion) separately for men and women from Nembro aged over 65. Moreover, we estimate the z-score for a fictional population that has 100 times the size of the Nembro population but the same probability of death. Thus, relative to the population size, the number of observed and expected deaths is equal. While in this setting, both the p-score and the PAF result in the same percentage of attributable deaths, the z-score is ten (i.e. $\sqrt{100}$) times higher in the fictional population (Table 2 B). This was a simplistic calculation. The methodology proposed by Farrington et al. [28] and applied by FluMOMO is more complicated. The dependence of the standard error on the sample size is accounted for by a power transformation which leads to z-scores that are approximately normal distributed. Nonetheless, in contrast to the p-score and the PAF, assumptions about the distribution are necessary to obtain an interpretable *estimand*. This further increases the difficulties of understanding the concept of the z-score in the context of attributable deaths. A detailed discussion about the merits of the z-score and a comparison to the p-score is provided by Aron and Muellbauer [12]. They also give further arguments why the use of the z-score is generally not recommended to compare attributable mortality of different populations.

All the estimands and our recommendation for their usage to compare the burden of COVID-19 are summarized in Table 3.

**Table 3:** Definition of six epidemiological measures used to quantify COVID-19 attributable mortality. ${\varvec{O}}$ is the number of observable deaths and ${\varvec{E}}$ the number of expected deaths

Definition	Formula	Interpretation	Appropriate for comparison of attributable mortality
Excess deaths (RD)	$O-E$	Total number of excess deaths	no
Per capita rate of excess deaths (RD_p.c._)	$(O-E)/n\times \mathrm{100,000}$	Excess deaths per 100,000 inhabitants	no
Standardized mortality ratio (SMR)	$\frac{O}{E}$	Ratio of observed to expected deaths	yes
p-score	$\frac{O-E}{E}$	Proportion of deaths above the expected level	yes
z-score	$\frac{O-E}{sd(O)}$	Number of standard deviations away from what is expected	no
Population attributable fraction (PAF)	$\frac{O-E}{O}$	Proportion of attributable deaths	yes

## Discussion

To evaluate and compare different political strategies for understanding the public health burden of COVID-19 and drawing conclusions for future pandemics, a fair comparison of attributable deaths between countries and different populations is indispensable. We discussed six measures of COVID-19 attributable mortality, namely the RD, the RD_p.c._, the p-score, the z-score, the SMR and the PAF. With an illustrative definition of the six measures, we explain how they relate to each other. Moreover, our multiple real and fictional data examples not only demonstrate their interpretation, but also reveal their strengths and weaknesses.

They especially show that reporting of the RD, the RD_p.c._ or the z-score alone can lead to a severely misleading comparison of attributable deaths between different populations. Although the per capita rate RD_p.c._ may seem intuitive and easily communicable to a lay audience, we do not recommend its reporting. Differences of the RD_p.c._ may not be due to the burden of COVID-19, but due to differences in the general mortality of the populations. Even if the goal is not a comparison of attributable mortality, it may be easily used as such by the readers. Moreover, the RD_p.c._ provides no additional information compared to the SMR, the p-score or the PAF. These latter measures are an important complement to routinely reported public health metrics such as the RD. They directly rank countries by the proportion of attributable deaths and thus allow for major conclusions on the different political strategies. Unfortunately, there is a substantial amount of articles that use the RD_p.c._ in clinical practice for the evaluation of preventive strategies.

The SMR, the p-score and the PAF are relative epidemiological measures that allow for a fair comparison of attributable mortality of different populations. Nonetheless, in contrast to the SMR and the p-score, the PAF is rarely used for burden quantification of COVID-19. As the PAF has been specifically designed for the purpose of quantifying attributable mortality and for evaluating public health prevention strategies, we highlight some of its advantages. First, as the SMR and in contrast to the p-score, the PAF is a well-known and well-investigated public health metric in epidemiological literature [6, 10, 11]. It has an intuitive interpretation as proportion of preventable deaths. By differentiating the total number of observed deaths by those that are attributable to COVID-19 and those that would have occurred irrespective of the pandemic, it explains the observed mortality. Moreover, the PAF does not exceed 100%. This is an advantage over the p-score if the number of excess deaths is large, which may lead to an inflation of the p-score. The existence of a benchmark makes it easier to evaluate large values. The PAF is therefore suitable not only for quantifying COVID-19 attributable mortality but also for communicating the burden of COVID-19 to a lay audience. Given the interpretational benefit of the PAF, we encourage its usage to quantify the burden of COVID-19. The PAF may provide an essential contribution to understanding and communicating the burden of COVID-19 and to fighting this pandemic. Finally, we highlight that the PAF can be further used to quantify the years of life lost (YLL) due to COVID-19 [30]. While attributable mortality merely counts the number of deaths due to COVID-19, YLL takes also the life span into account that would have been expected had COVID-19 not occurred. Thus, the death of a young person with a long expected life time is up weighted compared to the death of an elderly person with a shorter remaining life time. A discussion on estimation of YLL due to COVID-19 is given by Devleesschauwer et al. [31].

All of the six measures of attributable mortality are estimated with all-cause mortality statistics. These data are unbiased with respect to the potentially unknown prevalence of COVID-19 infections and can be used to quantify the absolute burden of the COVID-19 pandemic. However, all-cause mortality statistics are not sufficient to identify the number of deaths directly attributable to COVID-19 [2]. Moreover, deviations between deaths officially attributed to COVID-19 and estimated attributable deaths cannot be explained by false or misleading reporting alone. That is because the estimated attributable deaths include indirect deaths of non-COVID-19 infected individuals [2, 9]. Furthermore, not all patients that die with COVID-19 die due to COVID-19. Thus, the official COVID-19 death count likely contains individuals whose death was not preventable even if COVID-19 had been eliminated.

## Conclusion

Even if all-cause mortality data are not sufficient to identify the number of deaths that are directly attributable to COVID-19, estimates from all-cause deaths are essential to understand attributable mortality and the burden of COVID-19 in different regions and for different high risk populations [32]. We recommend that the number of excess deaths, RD, is always reported together with the PAF, the p-score or the SMR instead of a per capita rate RD_p.c._. Estimation of these measures from excess deaths is straightforward. For a complete picture of COVID-19 attributable mortality, quantifying and communicating its relative burden also to a lay audience is of major importance.

## Supplementary Information

**Additional file 1.**

**Additional file 2.**

**Additional file 3.**

## Data Availability

Code of the data analysis (Additional file 1 (USA) and Additional file 2 (Germany). Details on the literature search (Additional file 3).

## Abbreviations

RD: Risk difference, i.e. absolute number of excess deaths
RD_p.c._: Excess deaths per capita
p-score: Proportion of deaths above expected level
SMR: Standardized mortality ratio
z-score: Number of standard deviations away from what is expected
PAF: Population attributable fraction
YLL: Years of life lost
