# Supplementary material for: Quantifying and communicating the burden of COVID-19
Source: BMC Med Res Methodol. 2021 Aug 10;21:164. doi: 10.1186/s12874-021-01349-z (PMC8353440; doi:10.1186/s12874-021-01349-z)
Supplement: Supplementary file 1 — Additional file 1. [file 12874_2021_1349_MOESM1_ESM.html]

Estimation of the population attributable fraction for the United States


# Estimation of the population attributable fraction for the United States

#### Dr. Maja von Cube, Institute of Medical Biometry and Statistics, Faculty of Medicine and Medical Center, University of Freiburg

#### 3/10/2021

This is the R code for estimating the population attributable fraction using the public estimated weekly excess deaths from Weinberger et al. (Weinberger, Daniel M., et al. “Estimation of Excess Deaths Associated With the COVID-19 Pandemic in the United States, March to May 2020.” JAMA Internal Medicine (2020).).

Frequently updated excess deaths from the U.S. can be downloaded here: https://github.com/weinbergerlab/excess\_pi\_covid/tree/master/outputs/national\_and\_state\_summary.csv.

```
# obtain the data (https://github.com/weinbergerlab/excess_pi_covid)
USdata<-read.csv("national_and_state_summary.csv", header=TRUE)

# select only the necessary columns
USdata<-dplyr::select(USdata, state,week_start_date, week_end_date, excess_all_cause_deaths,
                      baseline_all_cause, all_cause_deaths, baseline_all_cause_upper,
                      baseline_all_cause_lower)

# Our time window for estimation starts on March 1, 2020.
USdata<-USdata[USdata$week_start_date>="2020-03-01",]

# Estimation of the PAF for the United States
USagg<-USdata[USdata$state=="US.agg" & !is.na(USdata$state),]
USagg$PAF<-cumsum(USagg$excess_all_cause_deaths)/cumsum(USagg$all_cause_deaths)
# cumulative excess deaths over calendar time
USagg$RD<-cumsum(USagg$excess_all_cause_deaths)

# Estimation of the PAF for each state separately
statesNames<-unique(USdata$state, na.rm=TRUE)
statesNames<-statesNames[!is.na(statesNames)]

agg.data<-lapply(statesNames,FUN=function(i){
  output<-USdata[USdata$state==i & !is.na(USdata$state),]
  output$PAF<-cumsum(output$excess_all_cause_deaths)/cumsum(output$all_cause_deaths)
  output$RD<-cumsum(output$excess_all_cause_deaths)
  return(output)
})
```

Plot the population attributable fraction and the cumulative excess deaths of the USA in total and for all states separately:

Create Figure 1 from the main manuscript:
