# Supplementary material for: Quantifying and communicating the burden of COVID-19
Source: BMC Med Res Methodol. 2021 Aug 10;21:164. doi: 10.1186/s12874-021-01349-z (PMC8353440; doi:10.1186/s12874-021-01349-z)
Supplement: Supplementary file 2 — Additional file 2. [file 12874_2021_1349_MOESM2_ESM.html]

Estimation of the population attributable fraction for Germany


# Estimation of the population attributable fraction for Germany

#### Dr. Maja von Cube, Institute of Medical Biometry and Statistics, Faculty of Medicine and Medical Center, University of Freiburg

#### 3/10/2021

This is the R code for estimating the population attributable fraction using death statistics from federal statistical offices.

Frequently updated all-cause death statistics from Germany can be downloaded here: https://www.destatis.de/DE/Themen/Gesellschaft-Umwelt/Bevoelkerung/Sterbefaelle-Lebenserwartung/Tabellen/sonderauswertung-sterbefaelle.html;jsessionid=77B24B7B084002D12E9394E0398B9FFD.internet8731?nn=209016.

In a first step, the data needs to be transformed into dataframe with one column for the date and further columns for the daily death counts of the different agegroups.

```
# read the data
grippe16<-read_excel("jahr16_KW.xlsx")
jahr20<-read_excel("jahr20_KW.xlsx")

##################
##### Data preparation
##################

grippe16$...54<-NULL
jahr20$`53`<-NULL
# transpose data set

grippe16_transpose <- as.data.frame(t(as.matrix(grippe16)))
rownames(grippe16_transpose) <- 1:nrow(grippe16_transpose)


grippe16_transpose <- row_to_names(grippe16_transpose, row_number = 1)
grippe16_transpose$Woche<-1:nrow(grippe16_transpose)

grippe16_transpose<-grippe16_transpose[-1]

grippe16_transpose[-16]<- dplyr::mutate_all(grippe16_transpose[-16], 
                                           function(x) as.numeric(as.character(x)))

# deaths among people older and younger than 65
grippe16_transpose$over65<-rowSums(grippe16_transpose[9:15])
grippe16_transpose$under65<-rowSums(grippe16_transpose[1:8])

ncol_grippe<-ncol(grippe16_transpose)

# cumulative deaths
#for(index in 2:ncol_grippe){
#  grippe16_transpose[,paste0("cumsum",colnames(grippe16_transpose[index]))] <- cumsum(grippe16_transpose[index])
  
#}

# 2020

# transpose data set

jahr20_transpose <- as.data.frame(t(as.matrix(jahr20)))
rownames(jahr20_transpose) <- 1:nrow(jahr20_transpose)


jahr20_transpose <- row_to_names(jahr20_transpose, row_number = 1)
jahr20_transpose$Woche<-1:nrow(jahr20_transpose)

jahr20_transpose<-jahr20_transpose[-1]

jahr20_transpose[-16]<- dplyr::mutate_all(jahr20_transpose[-16], 
                                            function(x) as.numeric(as.character(x)))

# deaths among people older and younger than 65
jahr20_transpose$over65<-rowSums(jahr20_transpose[9:15])
jahr20_transpose$under65<-rowSums(jahr20_transpose[1:8])


# If wanted, the prepared data can be stored
# write.csv(jahr20_transpose," covid19_transpose.csv")
# write.csv(jahr16_transpose," grippe16_transpose.csv")
```

Then, the p-score, the standardized mortality ratio and the population attributable fraction can be estimated.

```
jahr20<-jahr20_transpose
grippe16<-grippe16_transpose

jahr20$under65<-rowSums(jahr20[1:8])
jahr20$over65<-rowSums(jahr20[9:15])


grippe16$under65<-rowSums(grippe16[1:8])
grippe16$over65<-rowSums(grippe16[9:15])


grippe16$cumsumover65<-cumsum(grippe16$over65)
grippe16$cumsumunder65<-cumsum(grippe16$under65)


jahr20$cumsumover65<-cumsum(jahr20$over65)
jahr20$cumsumunder65<-cumsum(jahr20$under65)

# select the week (beginning of the pandemic)
jahr20R<- jahr20 %>% filter(Woche>=10) %>% 
  dplyr::select(Woche, over65, under65)

grippe16R<- grippe16 %>% filter(Woche>=10) %>% 
  dplyr::select(Woche, over65, under65)


# total population younger and older than 65


n_2015<-c(64130785,17300178)
n_2019<-c(64301159, 18090682)

# stratified

# SMR
SMR_over65_16R<-((cumsum(jahr20R$over65)/n_2019[2])/(cumsum(grippe16R$over65)/n_2015[2]))


SMR_under65_16R<-(cumsum(jahr20R$under65)/n_2019[1])/(cumsum(grippe16R$under65)/n_2015[1])

#PAF
PAF_over65_16R<-(cumsum(jahr20R$over65)/n_2019[2]-cumsum(grippe16R$over65)/n_2015[2])/
  (cumsum(jahr20R$over65)/n_2019[2])


PAF_under65_16R<-(cumsum(jahr20R$under65)/n_2019[1]-cumsum(grippe16R$under65)/n_2015[1])/
  (cumsum(jahr20R$under65)/n_2019[1])

# p-score
score_over65_16R<-(cumsum(jahr20R$over65)/n_2019[2]-cumsum(grippe16R$over65)/n_2015[2])/
  (cumsum(grippe16R$over65)/n_2015[2])


score_under65_16R<-(cumsum(jahr20R$under65)/n_2019[1]-cumsum(grippe16R$under65)/n_2015[1])/
  (cumsum(grippe16R$under65)/n_2015[1])

# adjusted
# PAF
PAF_over65_16Radj<-(cumsum(jahr20R$over65)/n_2019[2]-cumsum(grippe16R$over65)/n_2015[2])/
  (cumsum(jahr20R$over65)/n_2019[2])*cumsum(jahr20R$over65)/(cumsum(jahr20R$over65)+cumsum(jahr20R$under65))


PAF_under65_16Radj<-(cumsum(jahr20R$under65)/n_2019[1]-cumsum(grippe16R$under65)/n_2015[1])/
  (cumsum(jahr20R$under65)/n_2019[1])*cumsum(jahr20R$under65)/(cumsum(jahr20R$over65)+cumsum(jahr20R$under65))

# p-score
score_over65_16Radj<-(cumsum(jahr20R$over65)/n_2019[2]-cumsum(grippe16R$over65)/n_2015[2])/
  (cumsum(grippe16R$over65)/n_2015[2])*cumsum(grippe16R$over65)/(cumsum(grippe16R$over65)+cumsum(grippe16R$under65))


score_under65_16Radj<-(cumsum(jahr20R$under65)/n_2019[1]-cumsum(grippe16R$under65)/n_2015[1])/
  (cumsum(grippe16R$under65)/n_2015[1])*cumsum(grippe16R$under65)/(cumsum(grippe16R$over65)+cumsum(grippe16R$under65))


# SMR
SMR_over65_16Radj<-(cumsum(jahr20R$over65)/n_2019[2])/(cumsum(grippe16R$over65)/n_2015[2])*
                    cumsum(jahr20R$over65)/(cumsum(jahr20R$over65)+cumsum(jahr20R$under65))

SMR_under65_16Radj<-(cumsum(jahr20R$under65)/n_2019[1])/(cumsum(grippe16R$under65)/n_2015[1])*
  cumsum(jahr20R$under65)/(cumsum(jahr20R$over65)+cumsum(jahr20R$under65))


PAF_16R<-PAF_over65_16Radj+PAF_under65_16Radj
SMR_16R<-SMR_over65_16Radj+SMR_under65_16Radj
score_16R<-score_over65_16Radj+score_under65_16Radj
```
