# Supplementary material for: Quantifying and communicating the burden of COVID-19
Source: BMC Med Res Methodol. 2021 Aug 10;21:164. doi: 10.1186/s12874-021-01349-z (PMC8353440; doi:10.1186/s12874-021-01349-z)
Supplement: Supplementary file 3 — Additional file 3. [file 12874_2021_1349_MOESM3_ESM.doc]

**Supplementary Material**

**Title: Quantifying and communicating the burden of COVID-19**

Maja von Cube PhD (corresponding author)^1^, Jéan-Francois Timsit Prof.^2,3^, Andreas Kammerlander M.Sc.^4^, Martin Schumacher Prof.^5^

^1^ Institute of Medical Biometry and Statistics, Faculty of Medicine and Medical Center - University of Freiburg

Office: Ernst-Zermelo-Straße 1, D-79104 Freiburg, Germany

Postal address: Stefan-Meier-Str. 26, D-79104 Freiburg, Germany

Phone: +49/761/203-7704; Mail: [cube@imbi.uni-freiburg.de](mailto:cube@imbi.uni-freiburg.de)

^2^ UMR 1137 IAME Inserm/Université Paris Diderot, 16 Rue Henri Huchard, 75018 Paris, France

^3^ APHP Medical and Infectious Diseases ICU, Bichat Hospital, 46 Rue Henri Huchard, 75877 Paris, France

^4^ Institute for Economics, Department of International Economic Policy, University of Freiburg, Rempartstraße 10 - 16, D-79098 Freiburg, Germany

^5^ Institute of Medical Biometry and Statistics, Faculty of Medicine and Medical Center - University of Freiburg, Stefan-Meier-Str. 26, D-79104 Freiburg, Germany

Search strategy

Plattform: Pubmed

Date of search: 13.10.2020

Search terms: [“COVID-19” OR “SARS-CoV-2”] AND [“attributable deaths” OR “excess deaths” OR “attributable mortality” OR “excess mortality”] (n=159)

- Exclude articles from before 2020

- Exclude ABSTRACT OR DATA SET OR CORRECTION OR NEWS OR REFERENCE MATERIAL

Databases= WOS, BCI, BIOSIS, CCC, DRCI, DIIDW, KJD, MEDLINE, RSCI, SCIELO, ZOOREC Timespan=All years

Search language=Auto


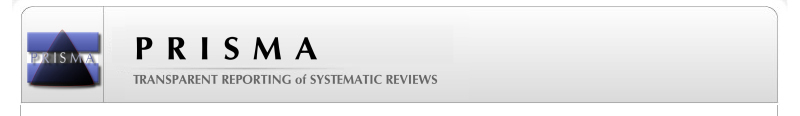
**PRISMA 2009 Flow Diagram**

**Screening**

**Included**

**Eligibility**

**Identification**

Records identified through database searching
(n = 147)

Additional records identified through other sources
(n = 0)

Records after duplicates removed
(n = 147)

Records screened
(n = 147)

Records excluded
(n = 112)

Full-text articles assessed for eligibility
(n = 35)

Full-text articles excluded, with reasons
(n = 0)

Studies included in qualitative synthesis
(n = 35)

Studies included in quantitative synthesis (meta-analysis)
(n = 35)
